# Comparison and risk factors analysis of multiple breast cancer screening methods in the evaluation of breast non-mass-like lesions

**DOI:** 10.1186/s12880-022-00921-3

**Published:** 2022-11-21

**Authors:** Jianxing Zhang, Lishan Cai, Xiyang Pan, Ling Chen, Miao Chen, Dan Yan, Jia Liu, Liangping Luo

**Affiliations:** 1 Department of Medical Imaging Center, The First Affiliated Hospital, Jinan University, No. 613, Huangpu Road West, Tianhe District, Guangzhou, 510630 Guangdong Province China; 2 Department of Ultrasound, The First Affiliated Hospital, Guangzhou University of Chinese Medicine, No. 16, Jichang Road, Baiyun District, Guangzhou, 510403 Guangdong Province China; 3 Department of Ultrasound, The Second Affiliated Hospital, Guangzhou University of Chinese Medicine, No. 111, Dade Road, Yuexiu District, Guangzhou, 510120 Guangdong Province China

**Keywords:** Breast ultrasonography, Non-mass-like lesion, Mammography, Automated breast ultrasonography

## Abstract

**Objective:**

To compare multiple breast cancer screening methods for evaluating breast non-mass-like lesions (NMLs), and investigate new best screening method for breast non-mass-like lesions and the value of the lexicon of ACR BI-RADS in NML evaluation.

**Methods:**

This retrospective study examined 253 patients aged 24–68 years who were diagnosed with breast NMLs and described the lexicon of ACR BI-RADS from April 2017 to December 2019. All lesions were evaluated by HHUS, MG, and ABUS to determine BI-RADS category, and underwent pathological examination within six months or at least 2 years of follow-up. The sensitivity, specificity, accuracy, positive predictive values (PPV), and negative predictive values (NPV) of MG, HHUS and ABUS in the prediction of malignancy were compared. Independent risk factors for malignancy were assessed using non-conditional logistic regression.

**Results:**

HHUS, MG and ABUS findings significantly differed between benign and malignant breast NML, including internal echo, hyperechoic spot, peripheral blood flow, internal blood flow, catheter change, peripheral change, coronal features of ABUS, and structural distortion, asymmetry, and calcification in MG. ABUS is superior to MG and HHUS in sensitivity, specificity, PPV, NPV, as well as in evaluating the necessity of biopsy and accuracy in identifying malignancy. MG was superior to HHUS in specificity, PPV, and accuracy in evaluating the need for biopsy.

**Conclusions:**

ABUS was superior to HHUS and MG in evaluating the need for biopsy in breast NMLs. Compared to each other, HHUS and MG had their own relative advantages. Internal blood flow, calcification, and coronal plane feature was independent risk factors in NMLs Management, and different screening methods had their own advantages in NML management. The lexicon of ACR BI-RADS could be used not only in the evaluation of mass lesions, but also in the evaluation of NML.

## Highlights

1. Ultrasonographic features of HHUS are significant for differentiating benign and malignant breast NMLs.
2. ABUS evaluation was associated with the highest sensitivity, specificity, PPV, NPV, and accuracy in the diagnosis and evaluating the risk of NML.
3. MG was superior to HHUS in specificity in diagnosis and evaluating the risk of NML.
4. Internal flow (HHUS), calcification (MG), and coronal plan feature (ABUS) may be risk factors for malignant or precursor lesions. The lexicon of ACR BI-RADS can also be used in the evaluation of NML.

## Introduction

As an effective method for early detection of breast cancer, mammography screening can reduce the mortality rate of breast cancer [1]. Ultrasound and automated breast ultrasonography (ABUS) is considered to be an important complementary method of breast cancer screening by mammography, especially for dense breast [2–6]. However, at least 25% of detectable breast cancers including the cases of non-mass-like lesions (NMLs) are missed by Mammography (MG) [7]. With the availability and widespread use of high-resolution breast US, the detection rate of breast NMLs has increased in recent years [8–10]. According to studies performed in Korea, the incidence of such lesions is estimated to be approximately 1–5.3% on screening breast US [9, 11]. To ultrasonography (US), NMLs refer to lesions that have no clear boundaries, no spatial mass effect on two or more different scanning directions [12, 13], and lesions that show ambiguous hypoechoic characteristics and architectural distortion without duct-like structure are usually defined as NML [14].

US and MG can detect mass-like abnormalities as standardized by the American College of Radiology Breast Imaging Reporting and Data System Atlas (BI-RADS) [15]. Currently, BI-RADS classification using ultrasound is not considered for detection of NML, and there are no standard NML guidelines. But studies have shown that these lesions have a more than 2% risks of malignancy [9, 10, 16]. Recently, studies based on B-mode, color Doppler flow imaging (CDFI), and strain or shear-wave elastography to distinguish benign from malignant NMLs have demonstrated their diagnostic performance [5, 14, 17, 18]. However, shear wave elastography was complementary to conventional ultrasound [18]. Similar to non-mass-like enhancement on breast MRI, with the enhanced image quality of high-resolution ultrasonography and widespread use of bilateral whole-breast examinations, it is possible to detect the hypoechoic regions (NMLs) that do not meet criteria for space-occupying lesions defined by BI-RADS [10, 16]. NMLs reflect a wide range of breast lesions, including benign, high-risk, and malignant lesions. High-risk lesions presenting as an NML include atypical ductal hyperplasia, lobular carcinoma in situ, papillary tumor, and squamous epithelial atypia. Squamous epithelial atypia is observed in NMLs, although absent squamous epithelial atypia has been reported [10].

There have been some studies on the relationship between US, NML lesion and their pathological importance [14, 19]. Ko et al. [14] classified breast NMLs into four categories based on calcification and architectural distortions, which are correlated with different BI-RADS categories. In previous studies, it was considered that ultrasound features were helpful in reducing biopsies of benign lesions [10]. However, our understanding of specific pathological features of NML that predict malignancy is incomplete in breast ultrasound. It is true that the methods used to evaluate breast US findings vary across practices and have been somewhat intuitive, and overlapping features make it difficult to accurately categorize breast NMLs, so tissue biopsy is required for classification.

Automated breast ultrasonography system (ABUS), also known as automated breast volume scanning, is a new imaging technology that can provide standardized image acquisition and coronal images of the entire breast. This system received FDA clearance as an auxiliary mean to screening mammography in 2008 [19]. By examining the breast continuously in transverse sections, ABUS can automatically perform three-dimensional breast reconstruction and simultaneously obtain morphologic and coronal image [6]. ABUS enhances the sensitivity, specificity and accuracy of breast lesion discrimination [20]. Previous studies of our group had also confirmed the value of ABUS in the re evaluation of high-risk lesions in mammography [21]. However, there was still little literature on the value of handheld ultrasound (HHUS) or ABUS in the diagnosis of breast NMLs. As we all know, different screening methods have their own advantages. However, there are few studies on the relationship among different screening methods, NML lesion findings and their pathological significance. This study compared and analyzed the evaluation of breast NMLs in three different breast cancer screening methods: mammography, HHUS, and ABUS. Our findings make contributions to the selection of effective screening methods for the detection of NMLs, and help to reduce the incidence of misdiagnosis and NML biopsy rate.

## Materials and methods

### Participants

**Fig. 1:**
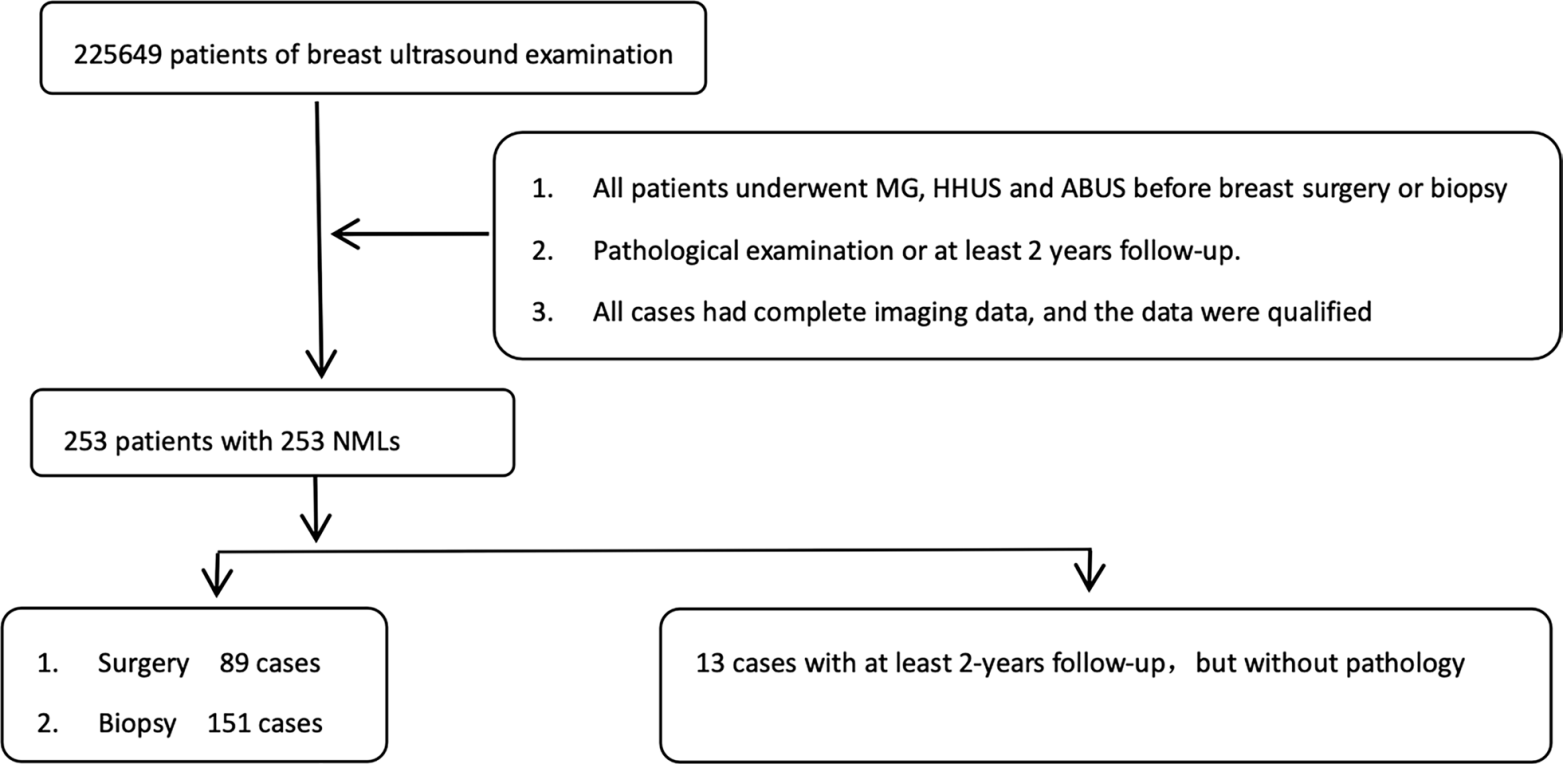
Flow chart showing follow-up and pathological results

This study was carried out in accordance with the Declaration of Helsinki and approved by the local institutional review board (ZE2020-232). The retrospective study examined 253 patients aged 24 to 68 years (mean age 45.33 ± 8.05 who was diagnosed with breast NMLs from April 2017 to December 2019. All patients underwent MG, HHUS, and ABUS before breast surgery or biopsy. We excluded those who did not undergo all 3 imaging studies or pathological examination or did not receive at least 2 years follow-up. Patients who had previously undergone surgery and pathological examination were also excluded (Fig. 1).

Only patients diagnosed with breast NML were included in our study. NMLs included ill-defined geographic hypoechoic or clustered hyperechoic spots without mass and tubular hypoechoic duct-like structural or architectural distortions on HHUS [13, 14]. These lesions exhibited suspicious calcification, distorted structure, asymmetry, and dilated ducts on mammography. In this study, 17 patients are younger than 35 years-age who had a family history of cancer or a high personal risk of cancer. 240 patients also underwent pathology within six months. 13 patients have benign NMLs, of which 5 cases were diagnosed according to breast MRI criteria (3 cases of BI-RADS category 1, 2 cases of BI-RADS category 3) and 8 cases were diagnosed by ultrasonography and ABUS (BI-RADS category 3). No change was found in the reexaminations of all 13 patients during the follow-ups in two years.

### Imaging acquisition and analysis

Mammography (MG) was performed using the Hologic-Lorad M-IV (Bedford, MA, USA). HHUS and ABUS were performed using the GE logiq E9 (GE, USA) with an ML6-15 liner probe at 10–14 MHz, Apollo 500 (CANON, JPN) and a PLT-1005BT liner probe at 10–12 MHz, and GE invenia ABUS (GE, USA) with a C15-6XW arc probe at 10 MHz. Preset ultrasonic instrument scanning conditions were used. Depth, gain and focus point was adjusted according to the thickness of the breast lesion area. The field of view was adjusted to include the area from the subcutaneous fat to the pectoral muscle layer.

The NML was located in the central region of the ultrasonography image, and two-dimensional longitudinal and transverse sections and CDFI were stored. All NML features were evaluated and recorded, including location, maximum diameter, echo pattern, structural distortion, ductal changes, microcalcification (hyperechoic, < 2 mm in diameter [22]), and posterior echo. To describe the distribution of microcalcification more accurately, scattered and aggregated point hyperechoic was used.

**Fig. 2:**
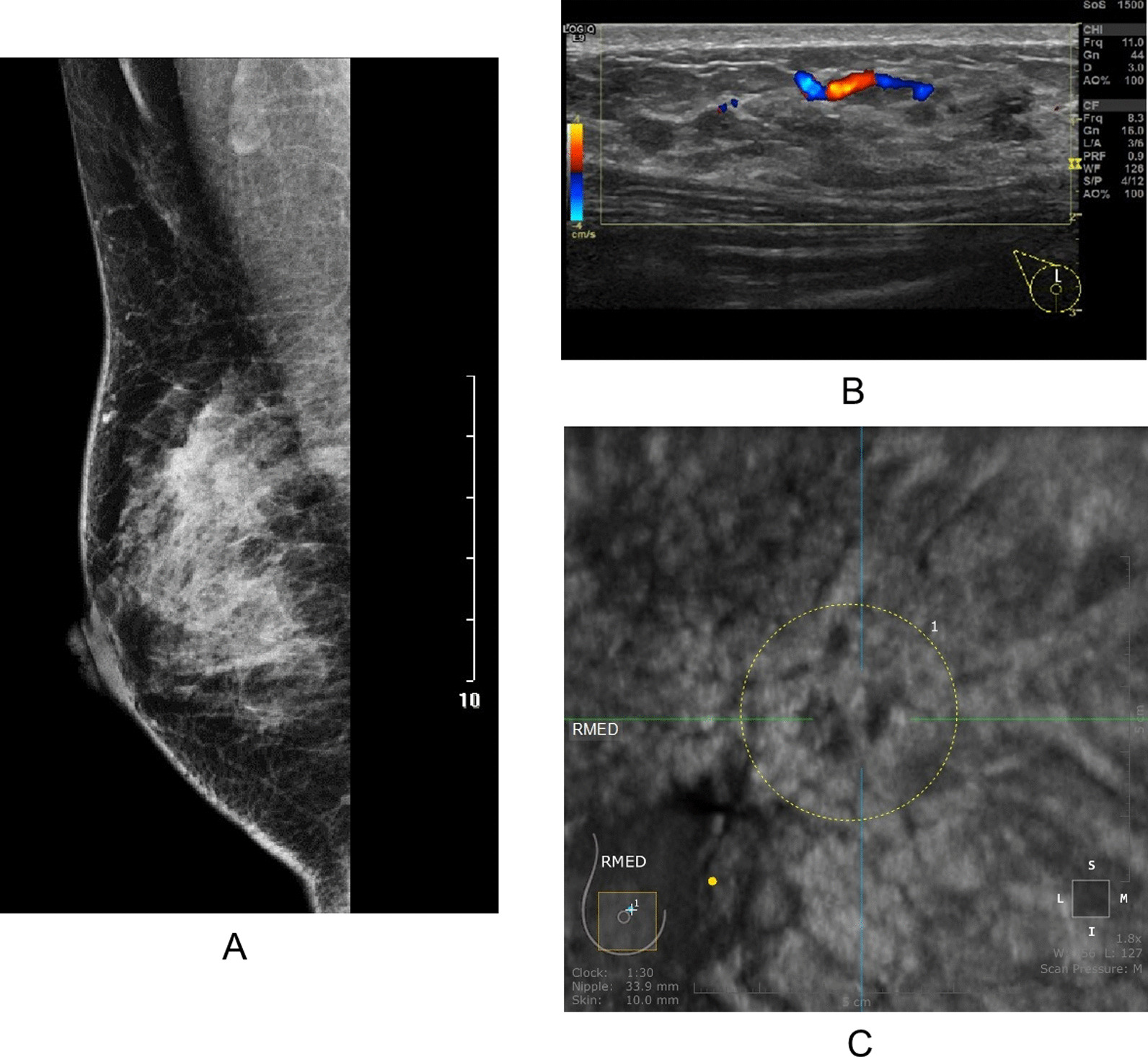
Hand-held ultrasonography and automated breast ultrasonography non-mass-like lesion findings with asymmetry (MG). **A** Large irregular block shadow with fuzzy and rough boundary and a large number of burrs like shadows of different lengths could be seen in the upper right breast area of MG, ACR BI-RADS 4A. **B** HHUS showed a patchy heterogeneous echo area in the right breast with irregular thickened duct structure. The thickened duct was hypoechoic. CDFI showed a little color blood flow signal in this area, Adler Grade 2, IA grade (K.O), ACR BI-RADS 4B. **C** The coronal plane of ABUS in the right breast showed patchy and uneven echo area with irregular shape, fuzzy edge and uneven internal echo. ACR BI-RADS 4B. Pathology:Intraductal carcinoma

**Fig. 3:**
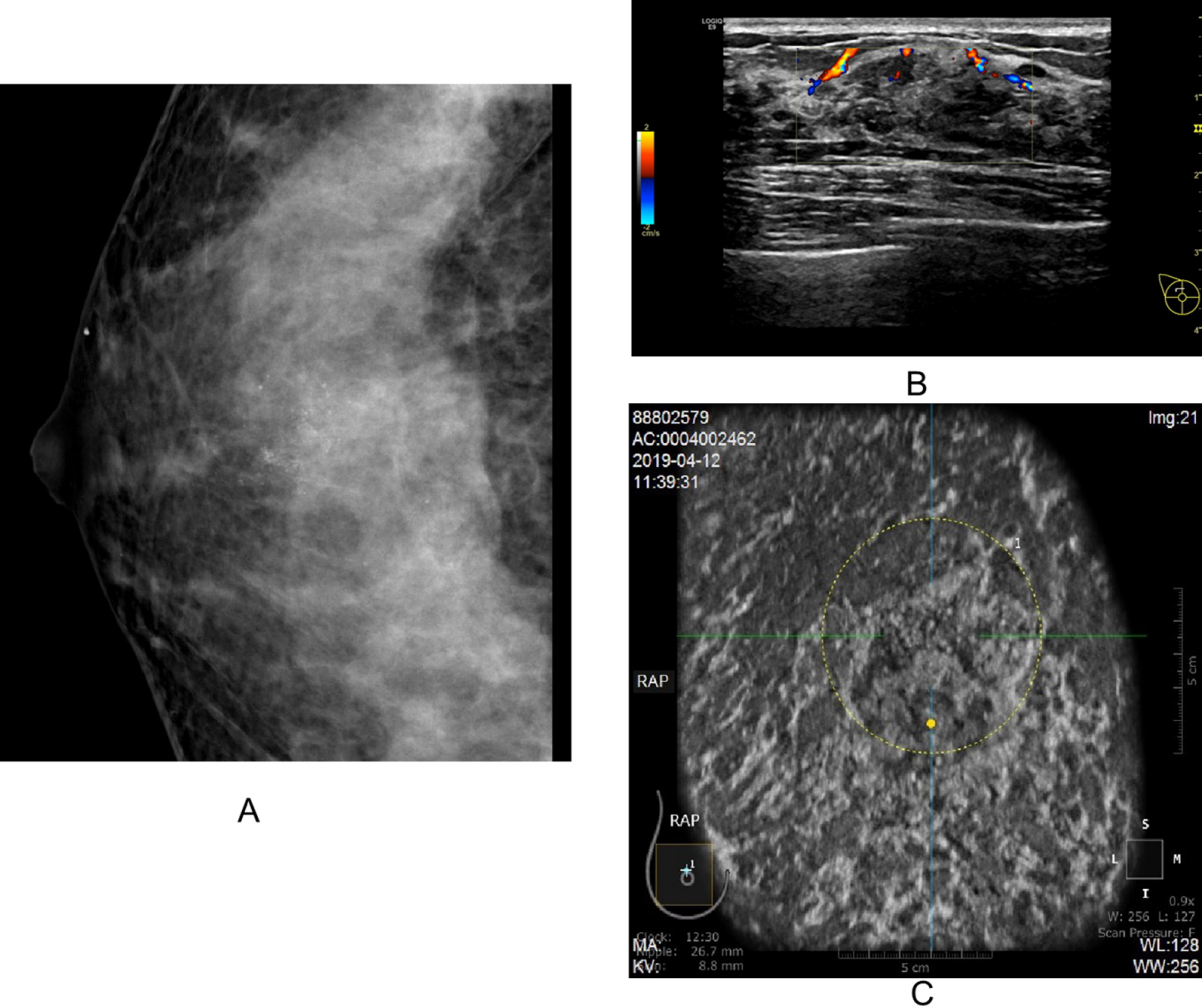
Hand-held ultrasonography and automated breast ultrasonography non-mass-like lesion findings with aggregated calcification (MG). **A** Mammography showed multiple punctate and irregular calcification in the upper part of the right breast without distortion of local structure and obvious mass sensation. ACR MG BI-RADS 4B. **B** HHUS showed patchy heterogeneous echo area in the right breast with disorder of the internal structure and multiple punctate hyperecho; CDFI showed strip blood flow signal in the heterogeneous echo area, Adler grade 1.ACR BI-RADS 4B. **C** ABUS showed patchy heterogeneous echo area in the upper right breast, with irregular coronal plane shape, irregular edge, local angulation, uneven internal echo and multiple punctate hyperechogenicity. ACR BI-RADS 5.Pathology: Breast invasive carcinoma (nonspecific type) with extensive intraductal carcinoma

**Fig. 4:**
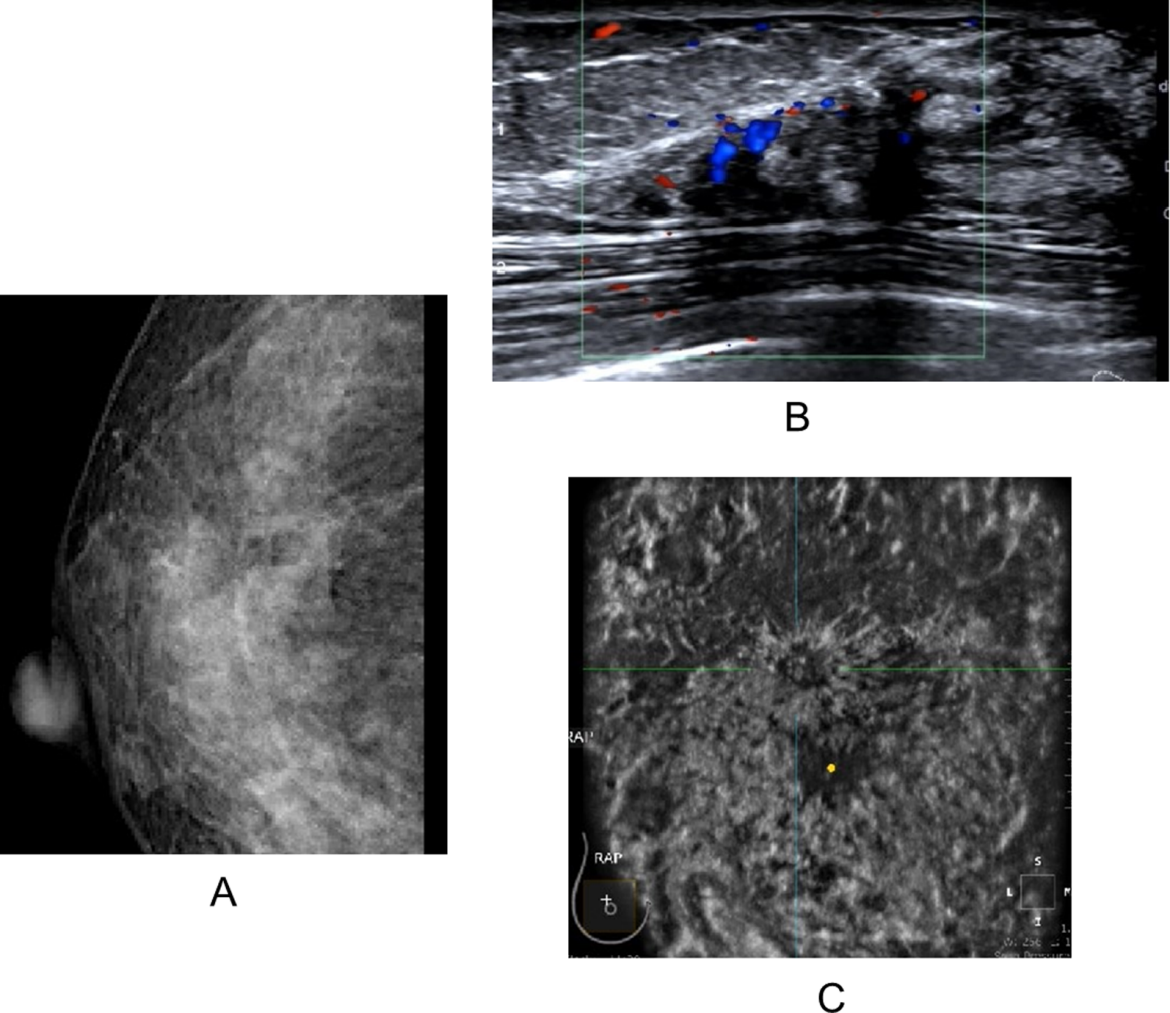
Hand-held ultrasonography and automated breast ultrasonography non-mass-like lesion findings with structural distortion (MG). **A** MG showed local structural distortion in the upper right breast without obvious mass and calcification, ACR BI-RADS 4A. **B** Ultrasound showed focal heterogeneous hypoechoic area with thickened ductal structure, slightly increased blood flow signal, Adler grade 3, ACR BI-RADS 4C. **C** ABUS showed hypoechoic mass on the coronal plane with irregular shape, irregular edge and convergence sign. ACR BI-RADS 5. Pathology: Invasive breast cancer (nonspecific type)

All NMLs were classified according to BI-RADS categories [13]. CDFI was evaluated according to Adler’s grade. The category in two-dimensional sonography was used as the reference for ABUS classification. However, if the coronal appearance of ABUS was consistent with mass, the lesions were classified according to the lexicon of ACR BI-RADS. All mammography and MRI features were evaluated using the lexicon of ACR BI-RADS (Figs. 2, 3 and 4).

All examinations were performed and analyzed by two radiologists (L.Z, with 20 years of mammography diagnosis and GY.Y, with more than 30 years of mammography diagnosis) or two sonographers (LS.C and L.C, with more than 15 years of breast ultrasound experience). The senior radiologist (LP.L. professor of imaging) acted as the third evaluator to solve the disputes of different doctors. Consistency of NMLs location was evaluated by two radiologists (M.C and JX.Z) according to mammography stereo localization method and targeted ultrasound localization method [21, 23]. Based on ACR BI-RADS guidelines, no high-risk was recommended in category 3 or category 2. High risk was recommended in those category 4 to 5, and biopsy or pathological examination was recommended [15].

### Histological characteristics

According to the 2019 World Health Organization classification of tumors of the breast [22], ductal carcinoma in situ (DCIS) and lobular carcinoma in situ (LCIS) are defined as breast precursor lesions. We included LCIS, DCIS, and other malignant tumors in the malignant and precursor lesions group. The benign lesions group included all other lesions, such as atypical ductal hyperplasia (ADH), flat epithelial atypia (FEA), and other benign lesions.

### Statistical analysis

Quantitative data are expressed as means with standard deviation and categorical data as composition or rate ratios. Quantitative data are compared using the t-test or *Kruskal–Wallis* test as appropriate. Categorical data are compared using the *chi-square* test or *Fisher's* exact test. Influencing factors are analyzed by unconditional stepwise logistic regression. Sensitivity, specificity, accuracy, positive predictive value (PPV) and negative predictive value (NPV) of MG, HHUS, and ABUS for differentiating breast NMs are calculated using final pathologic findings as the reference. Statistical analysis is performed using SPSS software version 22.0 (Chicago, USA). *P* < 0.05 was considered significant.

## Results

### Histological Characteristics of breast NML

Among the 253 study patients, 73 (28.9%) of the patients had lesions classified as malignant or precursor (age from 24 to 68, mean age 45.70 ± 9.10) and 180 (71.1%) as benign (age from 24 to 64, mean age 44.88 ± 6.51). The age of these two groups showed no statistical difference (*P* > 0.05). The histological characteristics are presented in Table 1. Among the 73 malignant or precursor lesions, the histological characteristics are invasive carcinoma in 12 (16.5%), invasive carcinoma with DCIS in 17 (23.3%), DCIS in 37 (50.7%), multiform lobular carcinoma in 3 (4.1%), multiform invasive lobular carcinoma in 1 (1.4%), and solid papillary carcinoma in 3 (4.1%). Among the 180 benign lesions, the histological characteristics are hyperplasia of the breast with calcification in 38 (21.1%), hyperplasia without calcification in 23 (12.8%), hyperplasia with apocrine metaplasia in 3 (1.7%), ADH in 22 (12.2%), FEA in 7 (3.9%), sclerosing adenosis in 22 (12.2%), radial scar in 4 (2.2%), papilloma in 18 (10.0%), adenomatous hyperplasia in 21 (11.7%), stromal pseudoangiomatous hyperplasia in 1 (0.6%), adenosis with infection in 5 (2.8%), granulomatous lobular mastitis in 3 (1.1%), and 13 cases (7.2%) are considered benign after 2 years of follow-up.

**Table 1:** Pathological findings in 253 breast non-mass-like lesions

Pathological diagnosis	
*Malignant or precursor*	
Invasive carcinoma (non-special type)	12
Invasive carcinoma with DCIS	17
DCIS	37
Multiform lobular carcinoma	3
Multiform invasive lobular carcinoma	1
Solid papillary carcinoma	3
hyperplasia with calcification	43
*Benign*	
hyperplasia without calcification	18
hyperplasia with apocrine metaplasia	3
Sclerosing adenosis	22
ADH	22
FEA	7
Radial scar	4
Papilloma	18
Adenomatous hyperplasia	21
Pseudoangiomatous hyperplasia of stroma	1
Adenosis with infection	5
Granulomatous lobular mastitis	2
Benign after follow-up	13

### Association between imaging features and histological characteristics

As showed in Table 2, there are significant differences between benign and malignant breast NMLs in the following characteristics: size, hyperechoicity, peripheral change, ductal changes, microcalcification, posterior echo, peripheral CDFI, internal CDFI, coronal plane feature (ABUS), calcification (MG), and structural (MG). These findings indicate that these imaging features acquired by different techniques can effectively predict pathological diagnosis.

**Table 2:** Association between imaging features and pathological diagnosis

	Total (n = 253)	Pathologically benign (n = 180)	Pathologically malignant or precursor (n = 73)	*χ*^2^	P
*Internal echo*					
Homogeneous	24	21	3	3.454	**0.063**
Heterogeneous	229	159	70		
*Hyperechoic*					
Non	105	86	19	13.093	**0.001**
Scattered spotty	83	48	35		
Aggregated spotty	65	46	19		
*Peripheral CDFI*					
Adler 0	163	139	24	67.550	** < 0.001**
Adler 1	50	33	17		
Adler 2	23	5	18		
Adler 3	17	3	14		
*Internal flow*					
Adler 0	161	139	22	65.131	** < 0.001**
Adler 1	49	30	19		
Adler 2	25	8	17		
Adler 3	18	3	15		
*Ductal change*					
Non	182	129	53	14.617	**0.001**
Duct ectasia (anechoic inside)	29	28	1		
Duct ectasia (Low-echo inside)	42	23	19		
*Peripheral change*					
Non	216	167	49	29.008	** < 0.001**
Architectural distortion	36	12	24		
*Structure distortion X-ray*					
Non	173	134	39	11.449	**0.003**
Distortion	38	20	18		
Disorder	42	26	16		
asymmetry					
Non	223	167	56	12.826	** < 0.001**
Focal asymmetry	30	13	17		
*Calcification*					
Non	53	48	5	14.107	**0.001**
Scattered spotty	120	75	45		
Aggregated spotty or cluster	80	57	23		
*Coronal plane*					
Non	106	99	7	60.580	** < 0.001**
Obscure	103	67	36		

*χ*^2^ means the value of chi-square test, P means the significant value of chi-square testn

### Evaluation of NMLs by different imaging techniques

As showed in Table 3, HHUS, MG, and ABUS all showed significant differences in evaluating the risk of NML (*P* < 0.01). Sensitivity in evaluating the risk for ABUS, HHUS, and MG was 98.6%, 95.9% and 84.9% respectively. Respective specificity was 53.9%, 30.6% and 42.2%. PPV was 46.5%, 35.9%, and 37.3%. Respect NPV was 99.0%, 94.8% and 87.4%. Respective accuracy was 66.8%, 49.4% and 52.2%. ABUS was superior in sensitivity, specificity, PPV, NPV, and accuracy in evaluating the risk of NML compared to MG and HHUS. MG was superior to HHUS in specificity, PPV, and accuracy. HHUS was superior to MG in sensitivity and NPV.

**Table 3:** Evaluation of NMLs by different imaging techniques

	Total (n = 253)	Pathologically benign (n = 180)	Pathologically malignant or precursor (n = 73)	*χ*^2^	*P*
*Mammography*					
Non high-risk	87	76	11	16.973	< 0.001
High-risk	166	104	62		
*HHUS*					
Non high-risk	58	55	3	20.558	< 0.001
High-risk	195	125	70		
*ABUS*					
Non high-risk	98	97	1	60.366	< 0.001
High-risk	155	83	72		
*Mammography*					
Benign	148	122	26	22.128	< 0.001
Malignant	105	58	47		
*HHUS*					
Benign	121	113	8	55.890	< 0.001
Malignant	132	67	65		
*ABUS*					
Benign	145	140	5	106.799	< 0.001
Malignant	108	40	68		

HHUS, MG, and ABUS also showed significant differences in diagnosis of NML (*P* < 0.01). The sensitivity of ABUS, HHUS and MG was 89.0% 64.4% and 93.2% respectively. The specificity was 62.8%, 67.8% and 77.8% respectively. The accuracy was 70.4%, 66.8% and 82.2% respectively. The PPV was 49.2%, 44.8% and 63.0% respectively. The NPV were 93.4%, 82.4% and 96.6% respectively. Compared with MG and HHUS, ABUS was superior in sensitivity, specificity, PPV, NPV and diagnostic accuracy. MG was superior to HHUS in specificity.

### Independent risk factors predicting malignancy or precursor breast NML

Taking benign and malignant tumors as the dependent variable Y (malignant or precursor (Y = 1), benign (Y = 0)), possible influencing factors are considered the independent variable Xi: internal echo, punctate hyperecho, peripheral CDFI (HHUS), internal CDFI (HHUS), catheter change, peripheral change, structural distortion (MG), asymmetry (MG), calcification (MG), coronal plane feature (ABUS). The results were analyzed by unconditional stepwise logistic regression. As showed in Table 4, the predictive coincidence rate of the regression model is 81.3%. The influencing factors, relative risk, and 95% confidence intervals (CIs) for breast malignancy or precursor lesions were CDFI (internal) for HHUS (odds ratio (OR) = 2.51, 95% CI 1.68–3.75), calcification for MG (OR 2.21, 95% CI 1.34–3.65), and coronal plan feature for ABUS (OR 3.90, 95% CI 2.23–6.82). These factors positively correlate with breast malignancy or precursor lesions and may be associated risk factors.

**Table 4:** Unconditional stepwise logistic regression analysis on influencing factors of breast cancer

Factors	B	S.E	Wald	P	Exp(B)	95% CI	
						Lower	Upper
Internal flow	0.920	0.206	20.020	< 0.001	2.508	1.677	3.753
Calcification	0.794	0.256	9.593	0.002	2.211	1.338	3.654
Coronal plane	1.361	0.285	22.730	< 0.001	3.899	2.229	6.822
Constant	− 6.782	0.950	50.973	< 0.001	0.001	–	–

## Discussion

Approximately 20%–25% of BI-RADS 4 breast NMLs with microcalcification are subsequently found to be malignant [24]. Although MG has been shown to reduce the incidence of breast cancer, its effectiveness on biopsy rate is still debated [25]. However, there is lack of an established classification system for breast NMLs detected by imaging. As far as we know, this study is the largest cases of image analysis on NMLs. In this study, we found that ABUS has the highest sensitivity, specificity, PPV, NPV, and accuracy in diagnosis and evaluating the risk of NML. MG was superior to HHUS in diagnosis and evaluating the risk of the NML in specificity. Compared to each other, HHUS and MG have their own advantages. Internal blood flow, calcification, and coronal plane feature is independent risk factors in identifying benign and malignant lesions.

NML was described in different categories in previous study [14]. However, these categories are subjective and there are differences between observers. The lexicon of ACR BI-RADS was used in evaluating the NMLs in this study. Conventional ultrasonography characteristics such as size, internal echo, hyperechoic spot, peripheral blood flow, internal blood flow, catheter changes, peripheral changes, as well as the coronal plane of ABUS are significant factors in the evaluation of breast NMLs and provide a theoretical basis for accurate ultrasonographic differentiation of benign and malignant lesions. MG is important in screening and diagnosing breast cancer. Its main aim is to identify densities, microcalcifications, and asymmetry [26]. Ultrasonography can assist in further characterization. Breast densities may be solid or cystic with smooth or irregular margins [27]. Microcalcifications may be focal or diffuse, and calcifications may be coarse or fine. Multifocal, fine calcifications are more likely to be malignant, whereas uniform, large, coarse calcifications are usually benign. Furthermore, they may be stable or increase over time [28], which is characteristic of NMLs. Proliferative NMLs with calcification are often classified as more dangerous and require biopsy. In this study, NMLs in 64 patients exhibited hyperplasia, including 43 cases of hyperplasia with calcification and 3 of hyperplasia with apocrine metaplasia and calcification. Among these, 23 were classified as high-risk NML.

Breast symmetry is often the most difficult to characterize, as it shows great variation between individuals as well as between the left and right breasts of the same individual and even between different breast quadrants. As the primary screening tool for breast cancer, the sensitivity of conventional MG is approximately 70% [29], and the sensitivity decreases with the increase in the quality of breast tissue assessed. Moreover, 76% of cancers are missed in women with dense breast tissue, while the overlap of normal breast tissue can lead to false-positives [30, 31].

ABUS allows non-invasive imaging of tissue using real-time sonography with high sensitivity, specificity, and accuracy [32]. At different strain levels, ABUS acquires different sonographic features of fat, normal glandular tissue, fibrous tissue, DCIS, and infiltrating ductal carcinoma [33]. Moreover, vascular distribution by CDFI provides data regarding blood supply [34]. CDFI can depict microvascularity and allows continuous dynamic Fobservation of microcirculation. In our study, abundant blood flow is more frequently observed in malignant NMLs, similar to existing studies [14]. These features allow ABUS to differentiate various mass lesions. In the evaluation of breast NML malignancy by HHUS and ABUS, most studies have reported higher sensitivity for ABUS but higher specificity and accuracy for HHUS [35], which is consistent with our findings. ABUS allows experts to focus on a lesion from three orthogonal sections at the same time and display the accurate spatial position of the lesion in real-time, which allows a thorough and detailed evaluation [36].

In previous studies, lower diagnostic sensitivity and specificity restricted the use of ABUS [37]. Therefore, it is critical to determine independent breast NML risk factors to enhance diagnostic capability. The logistic regression model that combined NML and internal flow and coronal surfaces detected by ABUS provided a more objective and accurate method for characterizing NML. However, age is also important (breast lumps), as showed by epidemiology [38].

There are several limitations based on our study. First, the differences between observers and the standardization of image storage are well-known imaging limitations. To reduce these. All imaging was retrospectively analyzed by two experienced experts while standardizing the scanning. The concise definition of descriptors was discussed, and an evidence-based consensus was reached. However, it remains necessary to standardize an accurate and specific definition of NML. Second, the study was conducted at a single institution and involved a small number of subjects, so the disease distribution was not necessarily generally representative. Further large-scale multicenter studies are warranted.

## Conclusion

In conclusion, conventional ultrasonographic (HHUS) characteristics, such as internal echo, hyperechoic spot, peripheral blood flow, internal blood flow, catheter change, and peripheral change, as well as coronal features (ABUS), structural distortion (MG), asymmetry (MG) and calcification characteristics (MG) which was described in the lexicon of ACR BI-RADS were significant in evaluating the risk of malignancy in breast NMLs. Internal blood flow (HHUS), calcification (MG), and coronal features (ABUS) might be risk factors for malignant or precursor lesions. ABUS evaluation significantly increases the sensitivity, specificity, PPV, NPV, and accuracy in diagnosis and evaluating the risk of NML. HHUS was distinctly superior to MG in sensitivity and NPV in determining malignancy, however, MG was superior in specificity, and the lexicon of ACR BI-RADS could be used not only in the evaluation of mass lesions, but also in the evaluation of NML.

## Data Availability

The datasets used and analyzed during the current study are available from the corresponding author on reasonable request.

## Abbreviations

ABUS: Automated breast ultrasonography
HHUS: Hand-held ultrasound
NML: Non-mass-like lesion
CDFI: Color Doppler flow imaging
BI-RADS: Breast Imaging Reporting and Data System Atlas
DCIS: Ductal carcinoma in situ
LCIS: Lobular carcinoma in situ
